# The Effect of the Secret Agent Society Group Program on Parent-Teacher Agreement Regarding Children’s Social Emotional Functioning

**DOI:** 10.3390/bs13040322

**Published:** 2023-04-10

**Authors:** Shannon Gasparro, Shannon Bennett, Katarzyna Wyka, Andrea Temkin-Yu, Andreas Damianides, Renae Beaumont

**Affiliations:** 1 Department of Clinical Psychology, St. John’s University, New York, NY 10065, USA; 2 Department of Psychiatry, Weill Cornell Medicine/New York Presbyterian, New York, NY 10065, USA; 3 CUNY Graduate School of Public Health, New York, NY 10065, USA; 4 Touro College of Osteopathic Medicine, New York, NY 10065, USA

**Keywords:** social processing, social skills, emotion regulation, youth, parent-teacher agreement, program, autism, Attention Deficit Hyperactivity Disorder, anxiety

## Abstract

Differences in social-emotional processing and functioning characterize children with Attention Deficit Hyperactivity Disorder (ADHD), Autism Spectrum Disorder (ASD), and Anxiety Disorders. These can contribute to difficulties forming friendships and secondary challenges such as academic underachievement, depression, and substance use in adolescence. To be optimally successful, interventions typically require parents and teachers to have a shared understanding of a child’s social-emotional needs and use consistent support strategies across home and school environments. However, research is yet to examine the effect that clinic-based programs have on parent-teacher agreement regarding children’s social-emotional functioning. To the authors’ knowledge, this is the first published study to explore this. A sample of eighty-nine youth (aged 8 to 12 years) with ASD, ADHD, and/or an Anxiety Disorder participated in the Secret Agent Society Program. The Social Skills Questionnaire and Emotion Regulation and Social Skills Questionnaire were administered to parents and teachers at pre-program, post-program, and six-month follow-up. Parent-teacher agreement was assessed at each time point. Pearson Product Moment correlations and intraclass correlations indicated that parent-teacher agreement on the measures of children’s social-emotional functioning improved over time. These findings suggest that clinic-based programs can contribute to key stakeholders developing a shared understanding of children’s social-emotional needs. The implications of these findings and directions for future research are discussed.

## 1. Introduction

Differences in social-emotional functioning relative to neurotypical peers are a common feature of children with Autism Spectrum Disorder (ASD), Attention Deficit Hyperactivity Disorder (ADHD), and Anxiety Disorders [1]. These differences can include challenges in accurately ‘reading’ social situations (attending to, perceiving, and interpreting others’ emotions and intentions), regulating one’s own emotions, and considering, deciding on, and/or implementing contextually appropriate responses. Lemerise and Arsenio [2] proposed that each step of social information processing is influenced by an individual’s trait or state emotional experience and emotion regulation. They note the role that an individual’s emotional state plays in what they attend to in a social situation, the attributions they make about others’ actions, and their ability to generate and select from a range of behavioral responses [2]. Each of these dimensions serves as a potential therapeutic target to reduce the risk of challenges associated with social-emotional problems (e.g., academic underachievement, depression and anxiety in adolescence, and drug use) [3].

To be successful, therapies and programs often require caregivers and school staff to be ‘on the same page’ with identifying goals for change and implementing strategies to support children’s use of emotion regulation and social skills at home and at school [4]. Such strategies often involve prompting skill usage at appropriate times and praising or rewarding children for applying learned skills, and may be incorporated into a formal student Individualized Education Plan (IEP) [4]. Under the Individuals with Disabilities Education Act (IDEA), all children with a disability are eligible for free, appropriate public education in the least restrictive environment. A central tenet of IDEA is the importance of collaboration between all members of a child’s support team, both within and outside of school, to provide individually tailored, integrated intervention programming [4]. While parent- and teacher-support of skill learning and application is often a desired goal of clinic-based interventions for youth, few therapy programs systematically involve both parents and teachers to support children’s skill learning and application across home and school settings [5]. Furthermore, studies typically fail to examine the impact of programs on parent-teacher agreement regarding children’s social-emotional skill profiles.

A review of the literature suggests that without specific strategies in place to facilitate effective parent-teacher communication and collaboration, the level of parent-teacher agreement on measures of children’s social-emotional and behavioral functioning is typically low to modest. This applies both to ‘typically’ developing children [3,6,7] and those with clinical diagnoses, such as Attention Deficit Hyperactivity Disorder [8] and Autism Spectrum Disorders [9,10]. Parent-teacher agreement is also typically lower on measures of children’s social skills (e.g., the Social Skills Improvement System) [7,11] and internalizing symptoms (e.g., the Strengths and Difficulties Questionnaire) [6], than externalizing behaviors (e.g., the Devereux Early Childhood Assessment) [10], which may be more apparent to adults in both home and school contexts.

Some argue that factors such as the situational specificity of certain child skills and behaviors and differences in parent- and teacher perspectives on what constitutes ‘normative’ social-emotional development contribute to the lack of parent-teacher agreement on child behavioral rating scales [7]. Nonetheless, Kuhfeld et al. [3] note the importance of addressing discrepancies in adult perceptions of students’ competencies, highlighting how adult perceptions shape children’s perceptions of themselves and influence their educational and career aspirations and attainment, especially in minority youth [12].

To the authors’ knowledge, no studies have been published that explore the impact of a clinic-based social-emotional skills training program on parent-teacher agreement of children’s social-emotional processing and functioning. The current study aimed to address this gap in the literature by examining the impact of the Secret Agent Society (SAS) group program on parent-teacher agreement on two reliable, valid, and freely accessible measures of children’s social-emotional functioning: The Spence Social Skills Questionnaire [13] and the Emotion Regulation and Social Skills Questionnaire [14].

The Secret Agent Society (SAS) [15] is a standardized group social skills program featuring a video game, nine weekly child group ‘club meetings’, concurrent group parent coaching sessions, and weekly teacher tip sheets. Numerous papers have been published supporting the effectiveness of SAS in improving children’s social-emotional functioning, particularly for autistic children without intellectual impairment [14,16]. The current study evaluated the effectiveness of SAS in improving the emotion-regulation and social skills of eight- to twelve-year-old children with a diagnosis of ADHD, an anxiety disorder, and/or ASD. The results are published elsewhere [17] and suggested that children’s emotion regulation and social skills improved from pre- to post-program relative to treatment as usual, with improvements maintained at a six-month follow-up. The program also appeared to be equally effective for children with ADHD, an Anxiety Disorder, ASD, or any combination of these conditions [17].

A unique dimension of SAS is the incorporation of specific elements to enhance parent and teacher engagement in the program (e.g., group parent coaching sessions, teacher tip sheets), and to support their communication and collaboration in monitoring and rewarding children’s social-emotional skill usage at home and school (e.g., a skill tracker form or app). Due to the incorporation of these elements, it was hypothesized that parent-teacher agreement on measures of children’s emotion regulation and social functioning would improve from pre- to post-program (10 weeks) and that this improvement would be maintained six months after the program ended.

## 2. Materials and Methods

The study was granted IRB Approval by the academic institution where it was conducted and is registered at https://www.clinicaltrials.gov/ct2/show/NCT02574273?term=Pilot+Trial+of+a+Social+Skills+Group+Treament+%28Secret+Agent+Society%29&draw=2&rank=1 under the title: Pilot Trial of a Social Skills Group Treatment (Secret Agent Society); registration number: NCT02574273.

### 2.1. Participants

Participants for the study were recruited from three psychiatry clinics at an academic medical center: two general child and adolescent outpatient psychiatry clinics, and one specialized Autism treatment center. Participants included 89 children, 68 males, and 21 females, aged 8 to 12 years old (M = 9.70, SD = 1.23), and their primary caregivers and teachers. The sample of teachers comprised elementary and middle school teachers from a major metropolitan area. Youth were included in the study if they met the following inclusion criteria: pre-existing diagnosis of ADHD, ASD, or an Anxiety Disorder from a treating clinician, stable on medication at study entry, aged between 8 and 12 years, demonstrated at least age-appropriate school performance and identified by a primary caregiver as having significant peer socialization challenges. Diagnoses of ADHD and Anxiety Disorders were confirmed with the ADIS-IV-C/P [18] during the intake interview for the study, and ASD and Social Pragmatic Communication Disorder diagnoses were verified using the ADOS-2 [19] and ADI-R [20] prior to study entry. On average, each child had 2.79 diagnoses, with ADHD being the primary (n = 46) diagnosis. Additional primary diagnoses of child participants included ASD or Social Pragmatic Communication disorder (n = 27) and Generalized Anxiety Disorder (n = 9). Participant demographic information is shown below in Table 1.

### 2.2. Measures

#### 2.2.1. Social Skills Questionnaire (SSQ) Parent and Teacher Forms

The parent and teacher forms of the SSQ [13] were used to assess children’s social skills. The Social Skills questionnaires each contain 30-items describing different social behaviors that have been shown to be important for children’s acceptance by peers [21]. Respondents are asked to rate each item on a three-point scale based on the child’s behavior over the past four weeks (*not true* (0), *sometimes true* (1), or *mostly true* (2)). Adding the scores of each item (e.g., “Shares things with other kids his/her own age” or “Makes eye contact appropriately with others during conversations”) yields a total score, with higher scores indicating greater social competence. The questionnaire items on the teacher form of the measure closely mirror those of the parent form.

Published research supports the psychometric properties of the parent and teacher forms of the SSQ (SSQ-P and SSQ-T) [13] and demonstrates the utility of the measure with a range of clinical populations, including children with social anxiety [22] and ASD [16]. The SSQ-P and SSQ-T have also been shown to be sensitive to capturing changes in social functioning over time [23,24]. Copies of the measure are available upon request from the senior author of this paper.

#### 2.2.2. Emotion Regulation and Social Skills Questionnaire (ERSSQ) Parent and Teacher Forms

The ERSSQ [14] assesses children’s competence in the specific emotion recognition, emotion regulation, and social skills that are taught in the SAS Program. The questionnaire consists of 27 items in the parent form and 25 items in the teacher form. The respondent rates the frequency with which a child engages in social behaviors (e.g., “Recognizes when other people are being sarcastic or teasing”) on a five-point Likert scale ranging from *never* (0) to *always* (4). After reverse scoring items 6 and 15 on the parent-form of the measure (ERSSQ-P) and items 5 and 13 of the teacher-form of the measure (ETSSQ-T), the total score is calculated by summing the individual item scores. Higher scores indicate greater social and emotion regulation skills. Published research supports the reliability and validity of the ERSSQ-P and ERSSQ-T [14,25], which are available upon request from the senior author of this paper.

### 2.3. Procedure

Families who were considered to be potentially eligible for the study were approached to participate by their treating clinician at one of the study sites. Families were given information sheets and a contact phone number to find out more about the study, and to schedule an intake interview if they appeared to meet the inclusion criteria. During the intake interview, a study team member described the study to the potential child participant and their primary caregiver and had them sign assent and consent forms. A trained clinician then administered the ADIS-IV diagnostic interview to both the child and the parent. After the ADIS-IV, those who were not eligible for the study were provided with referrals for other treatment options.

The caregivers of eligible participants were asked to complete a Demographic Questionnaire, the Social Skills Questionnaire-parent form (SSQ-P) [13], and the Emotional Regulation and Social Skills Questionnaire-parent form (ERSSQ-P) [14] in the waiting room of the clinic or at home if they were unable to stay due to time restraints. The caregiver was then given a teacher cover letter and teacher forms of the Social Skills Questionnaire (SSQ-T) and Emotion Regulation and Social Skills Questionnaire (ERSSQ-T) to give to their child’s teacher to complete. The SSQ-P and the ERSSQ-P were again completed in scheduled parent training sessions at post-treatment and at a six-month follow-up. Parents were asked to forward the SSQ-T and ERSSQ-T to their children’s primary teacher at the end-of-treatment and six-month follow-up for completion and return either directly to the clinicians via email or a stamped self-addressed envelope, or to clinicians via the parents.

This was a randomized controlled trial, with treatment as usual participants being offered the SAS intervention after a three-month waiting period. For this paper, participants’ data were pooled across groups to optimize the power of data analyses, as a number of children’s teachers did not complete and return their questionnaires. The SAS Program was delivered by psychiatry, psychology, and social work staff and trainees to groups of three to six children and their caregivers. All program facilitators were required to attend an in-person two-day facilitator training course and participated in weekly online group supervision sessions with the program author.

A random sample of 20% filmed child and parent sessions was checked for treatment fidelity relative to session checklists. This percentage of coded sessions is consistent with randomized controlled trials of other psychological interventions [26,27,28], where 13–20% of filmed sessions were reviewed. Based on the random sample of 20% filmed child- and parent-sessions that were coded, treatment providers were 74% reliable in delivering child sessions and 79% reliable in delivering parent sessions. For an effectiveness trial, this falls within acceptable limits [29], and is likely an underestimate of true fidelity ratings due to session activities being coded as “skipped” if they were not clearly seen or heard on film. Clinicians were more consistent in delivering psychoeducation and core skills training components of the child and parent sessions (e.g., discussing how the human body signals feelings of happiness, anger, and anxiety) than in delivering generic group procedural items (e.g., reviewing the agenda at the beginning of a session).

#### The Secret Agent Society (SAS) Program

SAS aims to improve children’s skills in reading and responding to social situations in ways that help to build and maintain positive peer relationships. The program uses an interactive computer game and spy-themed small group activities to teach children how to detect their own emotions and the emotions of others, introduces children to cognitive-behavioral therapy strategies for coping with anxiety and anger, and offers guidance in how to integrate relevant information (including contextual cues) to accurately read social situations (e.g., differentiating friendly joking from mean teasing; step-by-step formulae for skills such as self-advocacy, talking and playing with others, group work, and managing bullying). Skills are taught during nine weekly 90-min child club sessions (3–6 children, 1–3 facilitators) and simultaneous 45–60-min parent group meetings. See Table 2 for a summary of the skills taught in SAS.

After each club meeting, children are assigned “missions” (skills practice tasks), which they are to document in their computerized Secret Agent Journals. For example, for the Cracking the Conversation Code mission, they practice using their Conversation ‘Code’ steps to start, continue, and end conversations with peers, and self-evaluate what they did well and identify areas for improvement. Children can create ‘Scene Generator’ pictures of their mission completion, and type or voice record their responses to the Secret Agent Journal questions. These are reviewed with the program facilitator at the beginning of the next weekly club meeting.

In addition to the missions that aim to promote skill generalization to home and school, the program features a range of program elements to support parent-teacher engagement, communication, and reinforcement of children’s skill usage. These include weekly teacher tip sheets, which provide brief summaries of the skills that children learn in SAS Club Meetings and tips for how school staff can support them in using these skills in the classroom and playground. The tip sheets also include recommendations for how school staff can create more supportive and enriching learning environments for neurodiverse students and promote neurotypical peer acceptance and inclusion. A skill tracker form/app is provided for parents and school staff to check-in with children on when they used their target social-emotional skills each day and to exchange credits earned for home- or school-based rewards (e.g., screen time, collector cards, character figurines).

Weekly parent group meetings involve the program facilitator initially helping parents to troubleshoot any challenges that they have faced over the past week in supporting their children to use their SAS skills. Parents are introduced to the social-emotional skills that children are learning in the concurrent club meeting and are advised how to help their children use these skills during the coming week. Parent sessions involve a mix of didactic instruction, group discussion, and role plays.

### 2.4. Data Analyses

Parent-teacher agreement regarding children’s social-emotional functioning was assessed in two ways. First, Pearson Product-Moment correlations (r) were computed to assess the strength of the relationship between parent- and teacher-report measures at pre-program, post-program, and at a six-month follow-up. The following standard classifications for r values were used: r = 0.10 = small effect size, r = 0.30 = medium effect size, and r = 0.50 = large effect size. Next, intraclass correlations (ICC) were computed to assess the inter-rater agreement between parents and teachers. For ICC, the two-way random average measures with the absolute agreement model were used [30]. The following classifications of ICC values were used: ICC values < 0.40 = poor agreement, values between 0.40 and 0.59 = fair agreement, values between 0.60 and 0.74 = good agreement, and values > 0.75 = excellent agreement [31]. Finally, to optimize the sample size, the data included parent and teacher assessments after all the children received the SAS intervention, i.e., either during the randomized controlled trial phase of the study or after a three-month waiting period. The sample size at each time point reflects the number of children for whom data was available from both parent and teacher assessments. Due to the exploratory nature of the analyses and the small sample size for many, the alpha was set at 0.05 for each analysis.

## 3. Results

Data from both parent and teacher assessments was available for 71 out of 89 children (79.8%) at pre-program, 42 out of 89 children (47.2%) at post -program, and 14 out of 89 children (15.7%) at six-month follow-up. There were no differences in social-emotional processing and functioning at each time point between children with data and those with data missing for either parent or teacher assessments. Changes in children’s social-emotional functioning over time are summarized using descriptive statistics (see Table 3).

The results showed that the strength of the relationships (size and significance of the correlation coefficients) generally increased between the parent- and teacher-report versions of the SSQ and ERSSQ from pre-program to post-program to six-month follow-up (r range = 0.11 to 0.68; see Table 3). Similarly, intraclass correlations reflected the poor inter-rater agreement between parents and teachers on the ERSSQ and SSQ measures at pre-program (0.22 and 0.35, respectively), which improved to fair at post-program (0.50 and 0.48) and approached good at six-month follow-up (0.59 and 0.62; see Table 4).

## 4. Discussion

The present study explored changes in parent-teacher agreement on measures of social-emotional processing and functioning over the course of a group resilience program for children with ASD, ADHD, and/or an Anxiety Disorder: the Secret Agent Society (SAS). As hypothesized, parent-teacher agreement appeared to improve from pre- to post-program, with signs of further improvements at six-month follow-up for participants for whom data was available. This was likely due to the inclusion of program elements that specifically engaged and supported communication, psychoeducation, upskilling, and collaboration between parents and teachers, such as weekly parent coaching sessions, teacher tip sheets, and a home-school skill tracker monitoring and rewards system.

To the authors’ knowledge, this is the first study to explore the potential of a clinic-based psychosocial program to improve parent-teacher agreement on children’s social-emotional functioning, with prior research typically demonstrating low to modest agreement between parents and teachers on measures of children’s emotions, social skills, and behavior [3,6,11]. The findings speak to the potential value of community-based service providers using similar methods to engage with parents and educators to support them to share a common vision in supporting children’s social-emotional development and to provide individually-tailored intervention programming—a central tenet of the Individuals with Disabilities Education Act [4]. Adopting a collaborative ‘team around the child’ approach may not only help to optimize the effectiveness of psychosocial and educational supports and programs for children, but also help parents and professionals to feel mutually supported and less stressed in their efforts to help children reach their potential [4].

Due to the exploratory nature and methodological limitations of this study, caution is warranted in the conclusions that can be drawn from it. Firstly, because of the limited sample size, data was pooled across participants (all of whom were offered SAS at some stage of the randomized controlled trial). Therefore, comparisons could not be made between the SAS and treatment-as-usual conditions in improvements made over time in parent-teacher agreement on measures of social-emotional functioning. It is possible that improvements would have been observed over time without involvement in SAS, either due to children’s social-emotional skills spontaneously improving at home and at school, or parents and teachers using other methods, such as school Individualized Education Plan (IEP) meetings to develop a shared understanding of children’s social-emotional skill profiles. Findings from large-scale research studies suggest that such improvements are unlikely, with social skill development in children tending to follow a stable trajectory over time unless systematic home and school support strategies are implemented [32].

There are also major shortcomings to the representativeness of the data captured in this study. Demographic information about parents and teachers was lacking, and the sample of child participants was largely Caucasian. Furthermore, a significant number of teachers did not return measures at the end of the program and follow-up time points. Those teachers who did return questionnaires may have been more engaged in the program and positive in their attitudes towards collaborative approaches to supporting students’ wellbeing, contributing to the improvements in parent-teacher agreement on the social-emotional functioning measures seen over time. Furthermore, it is unclear what the significance and implications are of the improvements in parent-teacher agreement on measures of children’s social-emotional functioning. Do these contribute to overall improvements in parent-teacher relationships, and what is the impact on students, caregivers, and school staff more generally?

Future research also needs to examine the broader impact of collaborative care models for promoting student well-being and academic success, including a cost-benefit analysis of the added time and resources such approaches require. Stakeholder barriers to adopting and implementing collaborative support models in a sustainable manner should also be identified, including identifying practical strategies to address these. For example, anecdotal feedback from community clinicians and educators has suggested that having a range of methods for parents and teachers to access and use the SAS home-school skill tracker based on individual preference (e.g., app, printed, or electronic form) improves the sustainable adoption of this monitoring and reward tool.

An additional avenue for future empirical investigation is identifying what elements of programs such as SAS appear to be the most effective in enhancing parent-teacher engagement, upskilling, communication, and collaboration, and for whom. For example, in the current study, it was unclear whether the parent coaching sessions, teacher tip sheets, skill tracker, or other program elements made the greatest contribution to the seeming improvement in parent-teacher agreement on measures of social-emotional functioning over time, or if this varied according to certain stakeholder or child attributes.

Finally, and most importantly, it is critical to consider how an individual child’s perspective, goals, values, and preferences can best be identified and prioritized in collaborative program planning and implementation. In SAS, children are asked to identify their personal goals and the ‘powers they wish to boost’ both in the initial intake session and the first child group club meeting. These goals and values are revisited on an ongoing basis throughout the program and inform the individual tailoring of curriculum content and delivery. Although child self-report measures of social-emotional functioning in children with ADHD and ASD are often perceived as lacking validity due to children having limited self-awareness of their difficulties [33], from a neurodiversity perspective, supporting children to advocate for their own needs and preferences and making these the foundation for support planning is critical to respecting their rights and wellbeing. Thus, empirically examining how children’s perceptions of their social-emotional processing and functioning affect initial service planning and engagement, change over time in response to participation, and relate to parents’ and teachers’ perspectives, is crucial to improving the quality of the support services provided to neurodiverse populations.

## Figures and Tables

**Table 1:** Participant demographic information.

**Age (M, SD)**	9.70	1.23
**Gender (n, %)**		
Male	68	76.4
Female	21	23.6
**Race (n, %)**		
Caucasian	70	78.7
African American	4	4.5
Biracial	4	4.5
Asian	2	2.3
Other/Undisclosed	9	10.0
**Ethnicity (n, %)**		
Non-Hispanic		
Hispanic	84 5	5.6 94.4
**Special Assistance at School (n, %)**		
Yes	74	88.1
No	10	11.9
**Diagnosis type (n, %)**		
ADHD	46	51.7
ASD or Social Pragmatic Communication Disorder	27	30.3
Anxiety Disorder	9	10.1
Tic Disorder	3	3.4
Not Specified	4	9.5

Note: Race/Ethnicity was undisclosed for n = 5 participants. Information on Special Assistance was missing for n = 5.

**Table 2:** Summary of skills taught in the Secret Agent Society small group program.

Club Meeting Number	Skills Taught
1	Detecting emotions in others from facial expression, voice tone and body posture clues (social perception skills)
2	Identifying body clues and situations where you feel low, medium and high levels of anxiety and anger
3	Relaxation tools/gadgets—slow breathing and replacing unhelpful thoughts with more helpful alternatives
4	Other relaxation tools/gadgets—e.g. imagery, doing a physical activity; step-by-step social problem-solving formula
5	Steps for talking to others and self-advocating-the Conversation Code
6	Steps for coping with mistakes (the Damage Control Code) and playing with others (the Play Code) Introduction to the Challenger Board Game to role-play social skills
7	Steps for distinguishing friendly joking from mean teasing Continue playing the Challenger Board Game
8	Steps for coping with bullying
	Continue playing the Challenger Board Game
9	Steps for coping with confusion
	Skill review and future planning
	Finish playing the Challenger Board Game
3-month Follow-up	Skill review and future planning
6-month Follow-up	Skill review and future planningGraduation ceremony

**Table 3:** Correlation coefficients between parent- and teacher-report measures of children’s social-emotional functioning at pre-treatment, post-treatment, and at 6-month follow-up.

M (SD)			M (SD)	R	p-Value	n
Pre-treatment						
SSQ-P	35.08 (7.92)	SSQ-T	34.54 (11.59)	0.11	0.38	71
ERSSQ-P	51.65 (10.06)	ERSSQ-T	50.30 (15.25)	0.24	0.05 *	71
**Post-treatment**						
SSQ-P	38.04 (7.28)	SSQ-T	34.48 (11.34)	0.37	0.02 *	42
ERSSQ-P	62.27 (9.04)	ERSSQ-T	54.28 (15.76)	0.45	<0.01 **	42
**6-month Follow-Up**						
SSQ-P	45.29 (9.35)	SSQ-T	37.79 (12.50)	0.53	0.05 *	14
ERSSQ-P	68.61 (9.11)	ERSSQ-T	58.50 (17.40)	0.68	<0.01 **	14

Note: * *p* ≤ 0.05, ** *p* ≤ 0.01. Abbreviations: *SSQ-P and SSQ-T* Social Skills Questionnaire -Parent and Teacher; *ERSSQ-P and ERSSQ-T* Emotion Regulation and Social Skills Questionnaire -Parent and Teacher; *r* Pearson Product-Moment correlation, *M(SD)* Mean (Standard Deviation).

**Table 4:** Inter-rater agreement between parent- and teacher-report measures of children’s social emotional functioning at pre-treatment, post-treatment, and at 6-month follow-up.

Pre-Treatment		ICC	95% CI	n
SSQ-P	SSQ-T	0.22	(−0.25, 0.52)	71
ERSSQ-P	ERSSQ-T	0.35	(−0.05, 0.60) *	71
**Post-treatment**				
SSQ-P	SSQ-T	0.50	(−0.02, 0.76) *	42
ERSSQ-P	ERSSQ-T	0.48	(0.08, 0.73) *	42
**6-month Follow-Up**				
SSQ-P	SSQ-T	0.59	(−0.11, 0.86) *	14
ERSSQ-P	ERSSQ-T	0.62	(−0.09, 0.88) *	14

Note: * *p* ≤ 0.05. Abbreviations: *SSQ-P* and *SSQ-T* Social Skills Questionnaire -Parent and Teacher; *ERSSQ-P* and *ERSSQ-T* Emotion Regulation and Social Skills Questionnaire -Parent and Teacher; *ICC* intraclass correlation, *CI* confidence interval.

## Data Availability

Deidentified data from this study is available from the authors upon request.
